# Coupling Robot-Aided Assessment and Surface Electromyography (sEMG) to Evaluate the Effect of Muscle Fatigue on Wrist Position Sense in the Flexion-Extension Plane

**DOI:** 10.3389/fnhum.2019.00396

**Published:** 2019-11-01

**Authors:** Maddalena Mugnosso, Jacopo Zenzeri, Charmayne M. L. Hughes, Francesca Marini

**Affiliations:** ^1^Motor Learning, Assistive and Rehabilitation Robotics Laboratory, Robotics, Brain and Cognitive Sciences Department, Istituto Italiano di Tecnologia, Genoa, Italy; ^2^Department of Informatics, Bioengineering, Robotics and System Engineering, University of Genoa, Genoa, Italy; ^3^NeuroTech Laboratory, Health Equity Institute, San Francisco State University, San Francisco, CA, United States

**Keywords:** proprioception, surface electromyography, wrist, robot-aided assessment, muscle fatigue

## Abstract

Proprioception is a crucial sensory modality involved in the control and regulation of coordinated movements and in motor learning. However, the extent to which proprioceptive acuity is influenced by local muscle fatigue is obscured by methodological differences in proprioceptive and fatiguing protocols. In this study, we used high resolution kinematic measurements provided by a robotic device, as well as both frequency and time domain analysis of signals captured via surface electromyography (sEMG) to examine the effects of local muscle fatigue on wrist proprioceptive acuity in 16 physically and neurologically healthy young adults. To this end, participants performed a flexion/extension ipsilateral joint position matching test (JPM), after which a high-resistive robotic task was used to induce muscle fatigue of the flexor carpi radialis (FCR) muscle. The JPM test was then repeated in order to analyze potential changes in proprioceptive acuity. Results indicated that the fatigue protocol had a significant effect on movements performed in flexion direction, with participants exhibiting a tendency to undershoot the target before the fatigue protocol (−1.218°), but overshooting after the fatigue protocol (0.587°). In contrast, in the extension direction error bias values were similar before and after the fatigue protocol as expected (pre = −1.852°, post = −1.237°) and reflected a tendency to undershoot the target. Moreover, statistical analysis indicated that movement variability was not influenced by the fatigue protocol or movement direction. In sum, results of the present study demonstrate that an individual’s estimation of wrist joint displacement (i.e., error bias), but not precision (i.e., variability), is affected by muscular fatigue in a sample of neurologically and physically healthy adults.

## Introduction

Over the last half-century researchers have convincingly demonstrated the importance of proprioceptive acuity in the control and regulation of coordinated movements, motor learning, and error correction (Jeannerod, 1988; Schmidt and Lee, 2005). Proprioception^1^ is commonly divided into two modalities: joint position sense (JPS) and kinesthesia, where the former refers to the ability of the subject to perceive a presented joint angle (Proske and Gandevia, 2009), while the latter refers to the ability to perceive movements of the body (Hiemstra et al., 2001). JPS is the most commonly examined among the two modalities, with research demonstrating that JPS acuity is impaired after physical injury (Hertel, 2008), as well as in elderly individuals and chronic post-stroke patients (Hughes et al., 2015). Deficits in upper extremity proprioceptive function are said to arise from changes in both the central and peripheral nervous systems (CNS and PNS, respectively) (Good et al., 2001; Resnick et al., 2003; Quiton et al., 2007). Specifically, CNS changes include decreased gray matter volume in the frontal (Good et al., 2001; Quiton et al., 2007) and parietal lobes (Good et al., 2001; Resnick et al., 2003), and reduced activity in proprioceptive regions of the basal ganglia (Goble et al., 2012), both of which may contribute to declines in position sense across adulthood. Changes in the PNS that account for declines in proprioceptive function include increases in capsular thickness (Swash and Fox, 1974), decreases in muscle spindle sensitivity (Burke et al., 1996; Kim et al., 2007) and diameter size (Kararizou et al., 2005), as well as decreases in the number of intrafusal fibers (Swash and Fox, 1974) and cutaneous mechanoreceptors (Aydoğ et al., 2006).

Previous research has examined the effects of exercise-induced local muscle fatigue on JPS (Kazutomo et al., 2004; Givoni et al., 2007; Ribeiro and Oliveira, 2007; Ju et al., 2010; Karagiannopoulos et al., 2019; Sadler and Cressman, 2019) with somewhat mixed results. A previous study investigated the changes in knee JPS due to exercise-induced muscle fatigue (i.e., 30 consecutive maximal concentric contractions of the knee extensors and flexors) and reported an increase in both absolute and relative angular error in the extension direction after fatigue (Ribeiro and Oliveira, 2007). In contrast, another research failed to find differences in internal and external shoulder proprioception after a fatigue protocol in which participants performed two bouts of maximal reciprocal concentric isokinetic contractions until force output decreased below 50% of the participant’s maximum voluntary contraction (MVC) (Sterner et al., 1998).

One possible explanation for the divergent results lies in the different JPS protocols used in prior studies (Ager et al., 2017). For example, in previous protocols an experimenter passively moved the patient’s limb to a target position and then back to the starting position, after which the participant actively moved the same limb to the remembered position (Sharpe and Miles, 1993; Sterner et al., 1998; Kazutomo et al., 2004). Given that it is nearly impossible for the experimenter to maintain movement velocity across trials and participants, and that JPS is influenced by the speed of movement (Goble and Brown, 2009), this approach is considered to be a coarse measure of proprioceptive acuity.

With respect to measurements of end-point error, researchers have used techniques such as 2D motion capture based on video analysis (Kazutomo et al., 2004; Ribeiro et al., 2007) which have been found to be less reliable than isokinetic dynamometer and continuous passive motion devices (Ager et al., 2017). Moreover, the conflicting findings in prior studies may arise from the specific fatiguing protocol used to induce local muscle fatigue, as well as the way that fatigue is quantified. For example, many researchers have defined local muscle fatigue as the exercise-induced decline of the peak torque (Sharpe and Miles, 1993; Sterner et al., 1998; Kazutomo et al., 2004; Ribeiro and Oliveira, 2007). However, these studies did not use surface electromyography (sEMG) to confirm whether their fatigue protocols resulted in a decrease in the frequency of motor unit discharge (Dideriksen et al., 2012) and muscle conduction velocity (Enoka and Duchateau, 2008).

The benefit of using sEMG to quantify the physiological responses accompanying local muscle fatigue is that frequency domain analyses [e.g., Fast Fourier Transform (FFT)] are capable of reliably measuring the spectrum shift to lower frequencies while temporal parameters [e.g., Root Mean Square (RMS)] measure the typical increase in signal amplitude (Merletti and Parker, 2004; Cifrek et al., 2009; González-Izal et al., 2010). A second benefit of using sEMG, rather than peak torque, to measure fatigue, is its application to clinical rehabilitation settings. Post-stroke upper limb hemiplegia alters the neural strategies underlying force regulation, resulting in decreased voluntary muscle activation (Bowden et al., 2014), altered motor unit (MU) firing rates (McNulty et al., 2014), a reduced ability to modulate MU firing (Mottram et al., 2014) and abnormal MU recruitment patterns (Hu et al., 2013). As such, fatigue protocols in which participants are required to generate muscle force levels during the performance of maximal voluntary torque movements are not appropriate for stroke patients.

In recent years, collaborative work by our laboratories has focused on developing and testing robotic devices for upper extremity neurorehabilitation in healthy (Mugnosso et al., 2018), and neurologically impaired populations (Squeri et al., 2011; De Santis et al., 2015; Marini et al., 2017b). The advantage of robotic devices is that they have better diagnostic and prognostic precision than current clinical evaluation measures, resulting in a greater sensitivity to subtle differences in neurological status (Rinderknecht et al., 2016). However, in order to understand sensorimotor functions in these populations, it is important that we have a clear understanding of normative function in neurologically and physically healthy individuals. Such data can enable the comparison of measurement values during initial clinical assessment and at later periods in the rehabilitation life cycle.

As such, the aim of this study was to examine the effects of local muscle fatigue on wrist proprioceptive acuity using a robotic device specifically designed for human neuromotor control and rehabilitation (Masia et al., 2009) and ensuring the rise of muscle fatigue through frequency domain analyses captured via sEMG. To this end, sixteen participants first performed an ipsilateral wrist flexion/extension joint position matching (JPM) test, then a series of planar wrist flexion and extension movements while immersed in a viscoelastic force field that induced local muscle fatigue in the flexor carpi radialis (FCR) muscle, followed immediately by a second block of the JPM test. It is hypothesized that the fatigue protocol would lead to a decrease in JPS performance in the flexion direction. However, because the fatigue protocol targets the FCR, but not the extensor carpi radialis (ECR) muscles, it is hypothesized that there would be no change in proprioceptive acuity in the extension direction.

## Materials and Methods

### Participants

Sixteen neurologically and physically healthy right-handed individuals (seven males and nine females, mean age 27.6 ± 2.9 years) participated in the current study. Videogame use habits was assessed by querying the amount of time participants spend playing games on weekdays and weekends, and by calculating the average daily amount of game playing time. Overall, participants spent 2.83 ± 0.80 h/day playing video games, with no significant difference between male and female participants (2.90 and 2.78, respectively). The study was carried out at the Motor Learning, Assistive and Rehabilitation Robotics Laboratory of the Istituto Italiano di Tecnologia (Genoa, Italy) in accordance with the Declaration of Helsinki and the local ethical committee (Liguria Region: n. 222REG2015).

### Experimental Apparatus

**FIGURE 1:**
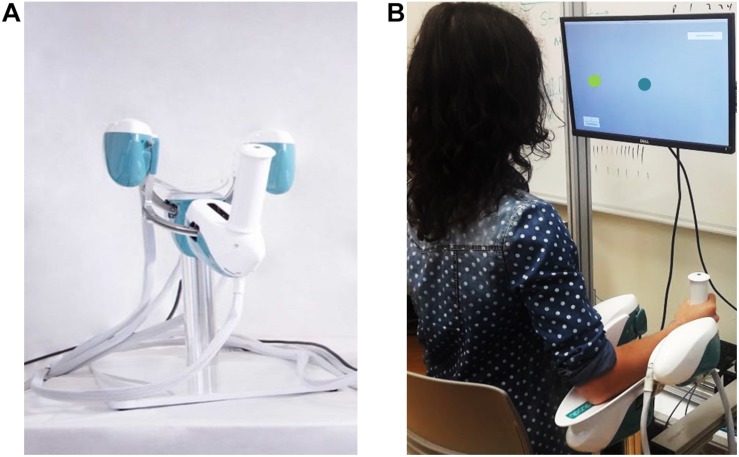
Experimental setup depicting the Wristbot **(A)** and a subject performing the Joint Position Matching test **(B)**.

The experiment involved the use of a haptic device [hereafter called Wristbot (Masia et al., 2009)] a 3 degrees of freedom (DoFs) fully backdrivable robotic manipulandum developed specifically for the study of human motor control and sensorimotor rehabilitation (Figure 1A). The Wristbot’s range of motion (ROM) in the three DoFs approximates that of the human wrist [flexion/extension (FE): human = 65°/70°, robot = 62°/62°; radial/ulnar deviation (RUD): human = 15°/30°, robot = 45°/40°; pronation/supination (PS): human = 90°/90°, robot: 60°/60°]. The Wristbot is powered by four brushless motors that provide accurate haptic rendering, and compensate for the weight and inertia of the device. The motors can provide a maximum torque of 1.57 Nm in the FE DoF, 3.81 Nm in the RUD DoF, and 2.87 Nm in the PS DoF. Angular rotations on the three axes are acquired by means of high-resolution incremental encoders with a maximum error of ±0.17°. A virtual reality environment was integrated into the system and provided users with visual feedback during the fatigue task.

A multichannel surface electromyography (sEMG) system (OTBiolab EMG-USB2+) was used to quantify activity of right extensor carpi radialis and flexor carpi radialis muscles (ECR and FCR, respectively) during the experiment. The sEMG system was set to collect data at 2048 Hz, with a gain of 1000, and a hardware bandpass filter (10–900 Hz). Following standard electrode preparation (Hermens et al., 1999), Ag/AgCl electrodes were placed on the ECR and FCR with an interelectrode distance of 26 mm. At the beginning of the fatigue task, a trigger signal was sent from the Wristbot to the sEMG base unit to ensure that the sEMG and Wristbot kinematic signals were correctly segmented and analyzed.

### Experimental Protocol

Prior to the experiment, participant provided written informed consent, after which his/her handedness was evaluated using the Edinburgh Handedness Inventory (Oldfield, 1971). Subsequently, the participant sat in front of the experimental setup, so that the participant’s body midline was vertically arranged with the computer monitor (Figure 1B), and grasped the Wristbot handle with his/her right hand. Then the experimenter ensured that the participant’s wrist axes were in correct alignment with the Wristbot, and used soft bands to strap the forearm to the mechanical support to ensure that the alignment would be maintained across the experimental protocol.

**FIGURE 2:**
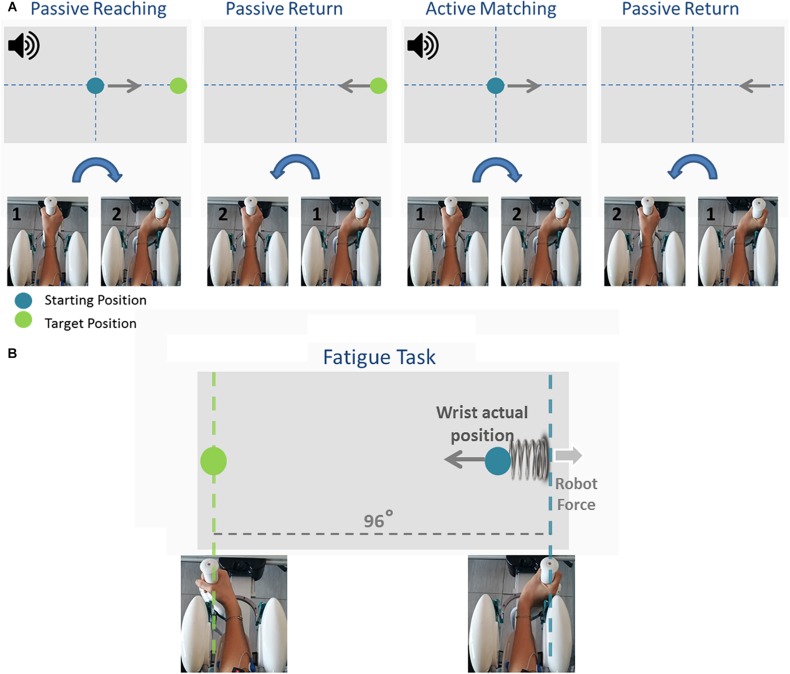
The temporal sequence of the Joint Position Matching task and the Fatigue task. During the Joint Position Matching task **(A)**, the robot moved the participant’s hand to a target location, held it there for 3 s, then brought the hand back to the start position. The participant then moved the robot handle to the remembered target position. During the fatigue task **(B)**, participants performed alternating flexion/extension movements, and the robotic device applied a viscoelastic force field that provided assistive forces to wrist extension movements and resistive forces during wrist flexion movements.

Participants first performed an ipsilateral Joint Position Matching (JPM) test (Figure 2A). Test instructions were explained to the participants, after which they performed 10 practice trials to familiarize themselves with the task and with the robot. Once the experimenter answered any of the participants’ questions, the participants’ vision was blocked with a pair of opaque glasses and the alignment of their wrist was rechecked. At the start of each trial, a high-frequency auditory cue sounded and the Wristbot moved the wrist from the start position (0°, neutral) to a determined angular position (passive reaching phase), and after 3 s brought the Wristbot handle back to the start position (passive return phase). A low-frequency auditory cue then sounded, and the participant moved actively the robot handle to the remembered target position and pressed the handheld response button when they believed they were in the correct position (active matching phase). The Wristbot then returned the robot handle to the start position (return phase). The targets were randomly presented at 48° of ROM in the FE DoF (with a random shift of ±0.5° to prevent learning effects). Participants performed 18 trials in the flexion DoF and 18 in the extension DoF, yielding a total of 36 initial JPM test trials.

Participants subsequently performed a series of planar wrist flexion and extension movements while immersed in a viscoelastic force field that induced local muscle fatigue in the FCR muscles. The targets were presented at 48° of FE ROM in an alternating fashion, and participants moved the cursor to the target position using the visual feedback of the hand position provided on the computer screen (Figure 2B). A speed constraint prevented subjects resting between trials, and in the event that the participant did not reach the target within 1.5 s, the color of the cursor changed from green to yellow. The applied viscoelastic force field provided assistive forces to wrist extension movements and resistive forces during wrist flexion movements (see Eq. 1):

(1) $$F=-k\,\left(\theta -\theta_{\text{eq}}\right)-b\,\dot{\theta }$$

where θ_eq_ = 48° is the virtual spring equilibrium angle, θ is the actual wrist position that moves at speed $\dot{\theta }$. *k* (=22.2N/rad for female and = 27.7N/rad for male subjects, respectively) and *b*(= 1.77*Ns*/*rad*) are the stiffness of the elastic force and the damping coefficient of the viscous contribution. The difference in the stiffness value between genders was based on the empirical literature demonstrating that female grip force values are 30% lower than male grip force values (Bäckman et al., 1995; Phillips et al., 2000), and enabled us to obtain comparable results for all participants. Participants were instructed to perform the fatigue task until the experienced forearm fatigue prevented them from flexing or extending the wrist. During the fatigue task, the experimenter verbally encouraged participants to sustain the task as long as possible to ensure the maximum level of subjective fatigue was reached (i.e., “completely fatigued” on the Borg CR-10 scale of perceived fatigue (Borg, 1990).

After the fatigue task, participants immediately completed a second block of the JPM test. The mean inter-task interval between the fatiguing protocol and the second JPM task was 45 ± 12 s.

### Data and Statistical Analysis

Robot encoders provided data at a 100 Hz sample rate which were used to extract angular displacements and angular velocities. The acquired data were processed with a sixth-order Savitzky–Golay low-pass filter (10 Hz cut-off frequency). Wrist proprioceptive acuity was evaluated using the metrics *Error Bias* and *Variability* (Schmidt, 1988; Dukelow et al., 2010; Marini et al., 2016a, 2018). *Error Bias* is defined as the average over the 18 trials, in each of the two directions (flexion and extension), of the difference between the reference joint angle (θ_*T*_) and the participants matching position (θ_i_) in the *i-*trial.

(2) $$E\,r\,r\,o\,r\,B\,i\,a\,s=\frac{\sum_{\mathrm{tr}=1:18}\left(\theta_{\text{i}}-\theta_{T}\right)}{18}$$

*Error Bias* provides information about participants’ response bias: it is the directional distance evaluated as algebraic summation between the ideal proprioceptive target and the actual wrist position, indicating the subjects’ tendency in undershooting (negative *Error Bias*) or overshooting (positive *Error Bias*) the target. *Variability* is defined as the standard deviation of matching position (θ_i_) across the 18 repetitions in each of the two directions (flexion and extension).

(3) $$V\,a\,r\,i\,a\,b\,i\,l\,i\,t\,y=\text{StD}\,\left(\theta_{1:18}\right)$$

While the *Error Bias* indicates error amplitude and is a direct measure of proprioceptive acuity and accuracy, the *Variability* measures the consistency across the 18 repetitions of the same target, thus providing information about precision.

**FIGURE 3:**
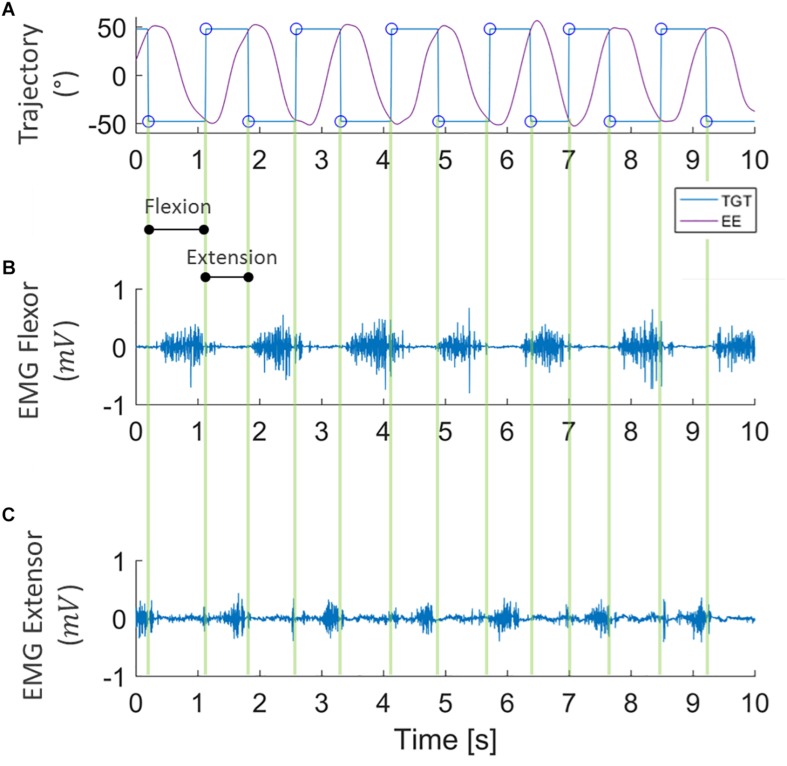
Example of data segmentation. Kinematic data collected from robot encoders **(A)** where purple lines represent the end-effector trajectory (EE) along flexion-extension to reach the target (TGT) that moves between ±48° (blue line). sEMG signal of *flexor* and *extensor carpi radialis* (**B**,**C**, respectively) during the task, segmented into flexion and extension directions.

To ascertain the occurrence of muscular fatigue, we first filtered the sEMG signals, recorded during the fatigue task, with a band-pass filter (5–350 Hz). The trajectory data was then extracted from the robot, and allowed us to determine the concentric phase of each movement. The sEMG signal of the FCR was analyzed during flexion movements, while ECR muscle activity was analyzed during extension movements. Indeed, as can be seen in Figure 3, the wrist kinematics recorded by the robot enabled us to segment the sEMG signal of the flexor and extensor carpi radialis during flexion and extension movements, separately.

For each movement, we then computed a single value of the *Mean Frequency* of the sEMG spectrum (Cifrek et al., 2009) using the formula:

(4) $$M\,e\,a\,n\,F\,r\,e\,q\,u\,e\,n\,c\,y=\frac{\int_{0}^{\frac{f\,s}{2}}f\,P\,\left(f\right)\,d\,f}{\int_{0}^{\frac{f\,s}{2}}P\,\left(f\,d\,f\right)}$$

where *fs* is the sampling frequency, and *P*(*f*) is the power spectrum density (PSD) of the signal. A Fourier transform of the autocorrelation function of the signal was employed to obtain a representation of the sEMG signal into the frequency domain, while the PSD was computed using the periodogram. Therefore, *N Mean Frequency* values were obtained for each subject, with *N* reflecting the total number of movements performed by the subjects.

The obtained *Mean Frequency* values were then fitted using a second order polynomial function based on mean least square approximation. To compare data among participants, *Mean Frequency* curves were interpolated separately for each participant, were normalized respect to the value of the first trial, and then averaged among subjects.

In addition to spectral analysis, we ensured the occurrence of muscle fatigue by examining the sEMG signal amplitude. Following the same segmentation and fitting procedures as above, we calculated the Root Mean Square parameter (*RMS*) on the filtered and rectified sEMG signal using the formula:

(5) $$R\,M\,S=\sqrt{\frac{1}{N}\,\sum_{i=1}^{N}x_{\text{i}}^{2}}$$

where *x*_i_ is the *i*^th^ sample of the sEMG signal, and *N* is the number of samples in the concentric phase of each movement.

Preliminary analysis did not reveal any systematic differences due to gender or video game experience. As such, potential differences effects of muscular fatigue on wrist proprioceptive acuity were examined using Repeated Measures Analysis of Variance (RM ANOVA) with Time (pre, post) and Direction (flexion, extension) as the within subjects factors, separately for the variables *Error Bias* and *Variability*.

## Results

**FIGURE 4:**
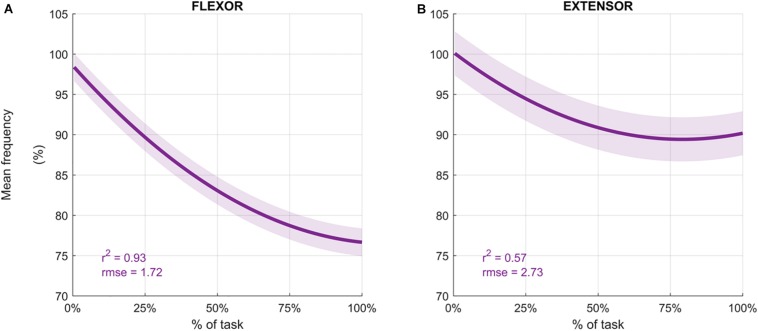
Average mean frequency values (%) for the flexor carpi radialis **(A)** and extensor carpi radialis muscles **(B)** during the fatigue protocol. Mean frequency (Hz) was normalized to the initial frequency of each sequence and averaged across participants to obtain the percentage. Depicted data was fitted with a second order polynomial function (dark purple) and standard error (light purple).

**FIGURE 5:**
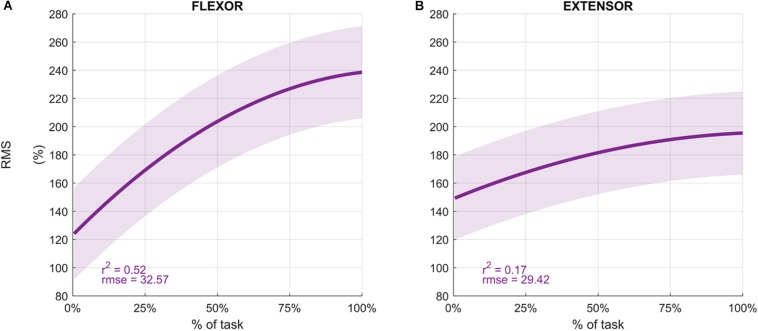
Average Root Mean Square values (%) for the flexor carpi radialis **(A)** and extensor carpi radialis muscles **(B)** during the fatigue protocol. RMS was normalized to the initial value of each sequence and averaged across participants to obtain the percentage. Depicted data was fitted with a second order polynomial function (dark purple) and standard error (light purple).

Participants performed an average of 146 ± 19 movements, during which the *Mean Frequency* of the sEMG signals decreased (Figure 4 depicts the normalized *Mean Frequency* curves for the flexor and extensor muscles (averaged across participants), with the corresponding goodness of the fit (*r*^2^) and *rmse*). Overall, the *Mean Frequency* of the flexor muscles exhibited a consistent decrease than that of the extensor muscles. Indeed, the average decrease in flexor muscle *Mean Frequency* was 25% of the original value (average *r*^2^ = 0.93), while extensor muscle *Mean Frequency* decreased by only 10% (average *r*^2^ = 0.57). The shift toward lower frequencies was accompanied by an increase in the signal amplitude, as shown by the *RMS* curves in Figure 5. Similarly, the rise of the *RMS* values was greater for the flexor than in the extensor muscles. Indeed, *RMS* reached 154% of the initial value in the flexor muscle (average *r*^2^ = 0.52), while *RMS* reached 98% in the extensor muscle (average *r*^2^ = 0.17).

**FIGURE 6:**
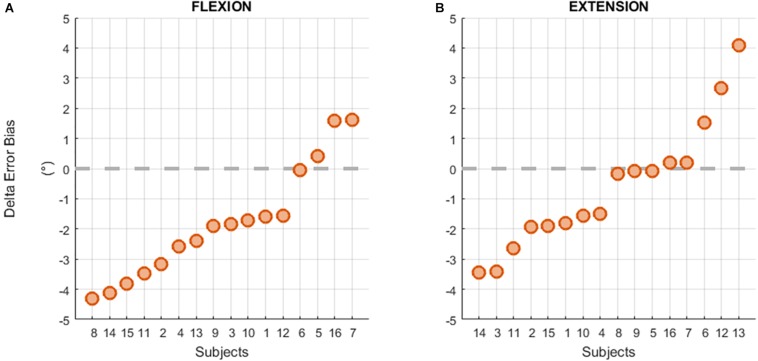
Individual differences between the *Error Bias* before and after the fatigue task in flexion **(A)** and extension **(B)** movements.

Individual results of the difference between the *Error Bias* after and before the fatigue task showed that 13 subjects out of 16 had a negative difference in the flexion direction (see Figure 6A) resulting from a greater overestimation of the reference position after the fatigue task. However, this trend was not present for targets located in the extension direction (Figure 6B).

**FIGURE 7:**
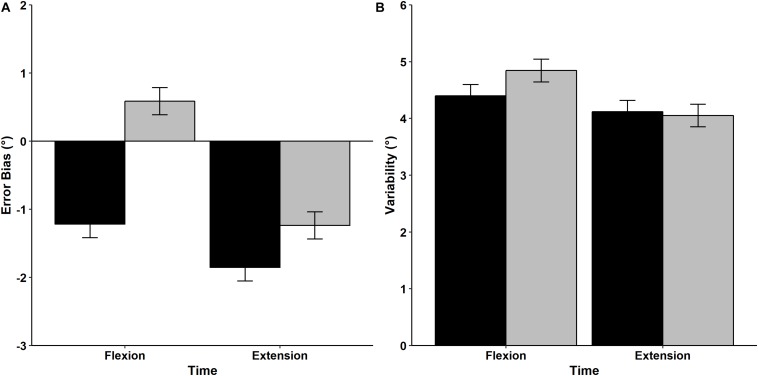
Mean and standard error values of *Error Bias* **(A)** and *Variability* **(B)** for movements performed in the flexion and extension directions before (black bars) and after (gray bars) the fatigue task.

Results of the mean *Error Bias* values prior to and after the fatigue protocol are displayed in Figure 7A. Analysis indicated that there was a significant main effect of Time, with participants exhibiting a greater tendency to undershoot the target prior to the fatigue protocol (−1.535°) compared to after the fatigue protocol (−0.325°) regardless from the direction, *F*(1,15) = 8.607, *p* = 0.010, η^2^*_p_* = 0.365. There was also a significant Time × Direction interaction, *F*(1,15) = 4.574, *p* = 0.049, η^2^*_p_* = 0.234. *Post hoc* analysis indicated that *Error Bias* values were similar before and after (pre = −1.852°, post = −1.237°) the fatigue protocol for the extension direction and reflected a tendency to undershoot the target. In contrast, the fatigue protocol had a significant effect on movements performed in flexion direction, with participants exhibiting a tendency to undershoot the target before the fatigue protocol (−1.218°), but overshooting after the fatigue protocol (0.587°).

Mean wrist proprioceptive acuity *Variability* is shown in Figure 7B. RM ANOVA revealed that mean *Variability* was similar regardless of direction [*F*(1,15) = 2.942, *p* = 0.107, η^2^*_p_* = 0.164] and time [*F*(1,15) = 1.221, *p* = 0.287, η^2^*_p_* = 0.075]. In addition, the interaction between Direction and Time was non-significant [*F*(1,15) = 0.945, *p* = 0.347, η^2^*_p_* = 0.059], indicating that movement *Variability* was not influenced by the fatigue protocol or movement direction.

## Discussion

This study utilized a high resolution robotic device and sEMG to examine the effects of local muscle fatigue on wrist proprioceptive acuity in healthy young adults. Congruent with prior research on the shoulder (Voight et al., 1996; Carpenter et al., 1998) and knee joints (Skinner et al., 1986; Lattanzio et al., 1997) we found that local muscle fatigue induces impairments in proprioceptive acuity. Prior research into this line of work used high-intensity isometric or isokinetic exercise protocols to elicit local muscle fatigue (Forestier et al., 2002; Lee et al., 2003; Walsh et al., 2004). However, we designed and implemented a fatigue task that involved concentric contractions restricted to the flexor muscles so that the impact of the fatigue task on the forearm extensor muscles could be minimized, and any potential damage to all forearm muscles could be avoided (Proske and Morgan, 2001). Utilizing this protocol, we were able to demonstrate that a high-resistive viscoelastic force field that targets only the FCR muscles is capable of eliciting a significant change in JPS response bias for the FCR, but not the ECR, muscle. Additionally, examining proprioceptive acuity in both flexion and extension muscles provided data regarding the repeatability of the measures. The lack of significant differences in proprioceptive acuity for the extension direction indicates that participants were equally focused on the task during the two repetitions of the test, thus ensuring that the changes in repositioning bias in flexion targets were entirely due to the fatigued FCR.

There is now a growing corpus of literature indicating that the muscle spindles are the primary receptor involved in the sense of position, whereas Golgi tendon organs, joint receptors, and skin receptors provide only limited information about joint movements throughout the normal range of motion (Proske and Gandevia, 2009). Muscle spindles are mechanoreceptors that are stretch-sensitive, and contribute to a person’s ability to perceive joint positions based upon information regarding the length and velocity of muscle contraction. As the muscle lengthens, there is a proportional increase in the discharge of the muscle spindles. Thus, the CNS can approximate the position of the limb based on the spindle firing rate and muscle length. However, when the muscle is fatigued, the high concentration of metabolites and inflammatory products of muscular contraction (e.g., bradykinin, arachidonic acid, prostaglandin E2, potassium, and lactic acid) causes the activation of nociceptors, greater alpha-gamma co-activation, and an increase in the muscle spindle discharge rate (Pedersen et al., 1998).

In the context of the current experiment, we attribute the changes in proprioceptive accuracy and precision to the differential impact that muscle fatigue exerts on muscle spindle discharge (Pedersen et al., 1998). Specifically, we hypothesize that muscle spindle discharge was affected by muscle fatigue, which resulted in the significant reduction in flexion direction proprioceptive accuracy. However, although affected, the muscle spindle discharge rate did not vary throughout the execution of the test. Consequently, the altered muscle spindle discharge rate did not prevent subjects from repeating similar errors in repositioning during the whole session as indicated by the absence of significant differences in *Variability* values across time. Therefore, the precise methods used in the present study not only clarifies conflicting findings from previous studies (Sterner et al., 1998; Kazutomo et al., 2004; Givoni et al., 2007), but also confirms that muscle fatigue decreases proprioception acuity by affecting the muscle spindles, but that muscle spindle discharge rate may not vary as long as the muscle is in a fatigued condition. The small but significant post-fatigue increase in *Error Bias* is congruent with prior studies that have reported a 1° difference in proprioceptive acuity, regardless of the examined joint [knee (Lattanzio et al., 1997) ankle (Forestier et al., 2001), elbow (Walsh et al., 2004)] and modality used to induce muscle fatigue (MVC or isokinetic movements). However, whether the magnitude of decrease in proprioceptive acuity is clinically relevant is still an open question (Refshaug, 2002), and the relationship between JPS acuity declines and alterations in motion or joint instability cannot be determined from the present work. The robotic device used in the present study was designed for use by both engineers and clinicians, and as such it is possible to adapt the task protocols to the needs of individual patients. In particular, the methodology of the present study may provide insights regarding proprioception sense in individuals that frequently experience muscle fatigue (e.g., individuals with neuromuscular disorders), as well as illuminate the extent to which symptoms of fatigue impact their motor control. In addition to the robotic systems relevance to populations with neuromotor dysfunction, the employed methodologies of the current study could add meaningful information regarding the prevention of injuries due to the fatiguing nature of manual work, or the maintenance of high level motor performance (i.e., in the case of athletes).

The efficacy of the fatiguing protocol used in the present study is supported by both the frequency and time domain analyses of the muscular signals captured via sEMG, in which a concurrent decrease in mean frequency and an increase in signal amplitude was observed. Time domain analysis indicated that a greater increase in *RMS* for the FCR muscle compared to the ECR muscle. Supporting this work, the frequency domain analysis revealed a 25% decrease in *Mean Frequency* for the FCR muscle, and 10% decrease in *Mean Frequency* for the ECR muscle, both of which are greater than the 8% decrease in *Mean Frequency* indicative of fatigue onset forwarded by prior research (Öberg et al., 1990). While at first glance (and according to the threshold proposed by Öberg et al., 1990) it may appear that the fatigue protocol elicited local muscle fatigue to both the wrist extensors and flexors, it is more likely that the muscle activation observed for the ECR is due to the co-activation of the FCR and ECR required to provide global stability to the wrist joint (Johansson et al., 1990; Myers and Lephart, 2000) and maintain smooth and even motions (Lieber and Lieber, 2002).

Another noteworthy point regards the locus of fatigue. It is well recognized that muscle fatigue can originate from central (e.g., insufficient drive from supraspinal sites, reflex inhibition, and disfacilitation) and/or peripheral mechanisms (e.g., decreased muscle fiber conduction velocity) (cf. Taylor et al., 2016). The observed decrease in EMG mean frequency, reflective of a decline in muscle fiber conduction velocity, indicates that the fatigue protocol resulted in peripheral muscle fatigue. However, we cannot conclusively state whether central muscle fatigue occurred in our study, as we did not employ the twitch interpolation technique or calculate the fractal dimension of the sEMG interference pattern (Beretta-Piccoli et al., 2015).

In evaluating the effects of local muscle fatigue on proprioceptive acuity, it is essential that the time interval between the execution of the fatiguing protocol and the following JPM test is minimized. Prior works used one device to test proprioceptive acuity and another one to implement the fatiguing protocol (Lattanzio et al., 1997; Walsh et al., 2004; Allen et al., 2007). For example proprioception of the knee joint has been measured using a JPM task where the participant sat on the end of a table with the knee at 90°, and the experimenter passively moved the participant’s limb to a target position and then back to the start position (Kazutomo et al., 2004). However, the local muscle fatiguing protocol required the participant sit on a Cybex isokinetic dynamometer and perform 60 consecutive maximum concentric knee flexion and extension contractions. We postulate that the lack of significant results found in different studies (Sharpe and Miles, 1993; Sterner et al., 1998; Kazutomo et al., 2004) may result from the amount of time that elapsed between the fatigue protocol and second proprioceptive acuity test. As such, a further novelty of the present work is the use of a robotic device that can both evaluate proprioceptive acuity and deliver the fatiguing protocol. In contrast to prior studies that had reported large intervals between the fatiguing protocol and the second JPM task (e.g., 15 min in Allen et al., 2007, 3 min in Lee et al., 2003, 5 min in Lattanzio et al., 1997), we were able to minimize the time between the execution of the fatigue task and the following JPM test to 45 ± 12 s, and as such avoided any potential muscle fatigue recovery between the two tests which could jeopardize the reliability of the JPM results. Furthermore, the use of the robotic device ensures the repeatability of the test and its high-resolution encoders guarantee the precision of the measures. Such advantages have already been exploited in previous works allowing to investigate the codification of proprioceptive information both in terms of kinesthesia (Marini et al., 2018) and joint position sense (Marini et al., 2016b, 2017a).

Our interest lies in understanding the neuromotor control mechanisms surrounding proprioceptive function, especially given that sensorimotor impairment in older adults is associated with recurrence of falls (Rossat et al., 2010) and a decline in the ability to perform functional activities (Shaffer and Harrison, 2007). It is likely that these deficits occur, in large part, due to the numerous anatomical and physiological changes that happen in the muscle spindle apparatus as people age (e.g., an increase in muscle spindle thickness (Swash and Fox, 1972), a decrease in intrafusal fibers and nuclear chain fibers (Jennekens et al., 1972; Swash and Fox, 1972; Lexell, 1992; Liu et al., 2005), and an increased proportion of type I extrafusal muscle fibers (Jennekens et al., 1972; Lexell, 1992). Further compounding this issue are changes in motor unit size, number, properties, and morphology that render older adults more prone to muscular fatigability compared to their younger counterparts (Kent-Braun et al., 2014; Hepple and Rice, 2016). Future research will aim to examine how local muscle fatigue impacts JPS acuity in aging populations, as well as determining factors (e.g., physical activity) that influence proprioceptive acuity in the aging population.

## Conclusion

In this study, we used a high resolution robotic device and frequency domain analyses of signals captured via surface electromyography (sEMG) to examine the effects of local muscle fatigue on wrist proprioceptive acuity in 16 healthy young adults. Utilizing a high-resistive robotic task to induce muscle fatigue of the flexor carpi radialis (FCR) muscle, we found that the fatigue protocol had a significant effect on movements performed in flexion direction, but not the extension direction. Thus, results of the present study indicate that an individual’s estimation of wrist joint displacement (i.e., *Error Bias*), but not precision (i.e., *Variability*), is affected by muscular fatigue.

## Data Availability Statement

Raw data were generated at the Motor Learning, Assistive and Rehabilitation Robotics Laboratory of the Istituto Italiano di Tecnologia. Derived data supporting the findings of this study are available from the corresponding author, MM, on request.

## Ethics Statement

The studies involving human participants were reviewed and approved by the local ethical committee (Liguria Region: n. 222REG2015). The patients/participants provided their written informed consent to participate in this study.

## Author Contributions

FM, MM, and JZ designed the experiment and formulated the experimental question. MM programed the robot and collected the data. FM, MM, and CH performed the data analysis and statistics and wrote the manuscript. JZ revised the final version of the manuscript.

## Conflict of Interest

The authors declare that the research was conducted in the absence of any commercial or financial relationships that could be construed as a potential conflict of interest.

## Footnotes

1 Although there is some debate over the concept of proprioception, we adopt the terminology used by Ribeiro et al. (2007) who defined proprioception as the cumulative neural input to the central nervous system from specialized mechanoreceptors located in the joint capsule, ligament, muscle, tendon, and skin.
